# A Primary Pancreatic Hydatid Cyst With Concomitant Hepatic Involvement Mimicking Mucinous Cystadenoma: A Case From an Endemic Region

**DOI:** 10.7759/cureus.94650

**Published:** 2025-10-15

**Authors:** Bouchra Hamade, Imad Semaan, Hayab Karaki, Rached Radwan, Abbas Rachid, Abbas Bahr

**Affiliations:** 1 Gastroenterology and Hepatology, Lebanese University, Beirut, LBN; 2 Internal Medicine, Saint Joseph University of Beirut, Beirut, LBN; 3 Medicine, Faculty of Medical Sciences, Lebanese University, Beirut, LBN; 4 Internal Medicine, Lebanese University, Beirut, LBN; 5 Gastroenterology and Hepatology, Bahman University Hospital, Beirut, LBN

**Keywords:** cyst, cystic pancreatic tumor, mucinous cystadenoma, pancreatic cyst management, pancreatic hydatid cyst

## Abstract

Pancreatic hydatid disease is an exceptionally rare manifestation of *Echinococcus granulosus* infection. Its clinical and radiological resemblance to other cystic pancreatic lesions often delays diagnosis and treatment. Here, we describe a case of a 71-year-old woman from Lebanon, who presented with progressive epigastric and right upper quadrant pain. Initial endoscopic examination revealed a benign gastric ulcer. Six months later, she developed obstructive jaundice and cholangitis. Examination showed scleral icterus and mild right upper quadrant tenderness. Laboratory tests revealed leukocytosis, markedly elevated transaminases, cholestatic enzyme elevation, and hyperbilirubinemia, with elevated C-reactive protein (CRP), consistent with acute cholangitis. Imaging identified cystic lesions in the pancreatic head and liver, initially suggestive of mucinous cystadenoma. Endoscopic retrograde cholangiopancreatography (ERCP) with biliary stenting relieved symptoms, but follow-up imaging demonstrated progressive lesion enlargement. Hydatid disease was suspected, given the concomitant hepatic cyst and endemic background. Surgical exploration confirmed cystic lesions in both organs, and histopathology established *E. granulosus* infection. The patient underwent Roux-en-Y cystojejunostomy and completed a postoperative course of albendazole without complications. Hydatid disease should be considered in the differential diagnosis of pancreatic cystic lesions in endemic areas, particularly when hepatic cysts coexist. Early recognition and combined surgical-medical management are essential to prevent complications and recurrence.

## Introduction

Cystic echinococcosis (hydatid disease) is a parasitic zoonosis caused by the larval stage of *Echinococcus granulosus* [1]. When the pancreas is affected, cysts are most often located in the head of the gland (in approximately 57% of cases), followed by the body (24%) and tail (19%) [2].

Humans become accidental intermediate hosts through ingestion of parasite eggs, typically via contaminated food or water, or through direct contact with infected definitive hosts. Once ingested, the larvae migrate and develop into hydatid cysts, most commonly in the liver (50-70% of cases), but they may also involve the lungs, kidneys, spleen, and, rarely, the central nervous system [3]. Hydatid disease is endemic in several temperate regions, including the Mediterranean basin, Central Asia, parts of China, North and East Africa, and South America [4].

The pathogenesis of pancreatic echinococcosis is not fully understood, though hematogenous dissemination is considered the most likely route. Other proposed mechanisms include lymphatic spread, biliary migration, or retroperitoneal extension [4]. Clinical manifestations are often delayed, emerging when the cyst enlarges sufficiently to compress adjacent structures. Common symptoms include epigastric or right upper quadrant pain, nausea, vomiting, and signs of biliary obstruction. Involvement of the pancreatic head may lead to obstructive jaundice and secondary cholangitis. Additional complications such as cyst rupture or superinfection have also been reported [5].

Diagnosis can be challenging due to the rarity of pancreatic involvement and the nonspecific nature of symptoms. Hydatid cysts may closely mimic other pancreatic cystic lesions, particularly mucinous cystadenomas, making radiological differentiation difficult, especially in uncomplicated or early-stage disease. Moreover, serological assays, while helpful, can yield false-negative results, particularly in isolated pancreatic disease [6].

Here, we report a rare case of pancreatic hydatid disease in a 71-year-old woman from Lebanon, an endemic area for echinococcosis. This case underscores the importance of considering hydatid disease in the differential diagnosis of pancreatic cystic lesions, particularly in endemic regions where atypical presentations may represent a parasitic origin. To our knowledge, the present study is one of the very few reported cases of synchronous hepatic and pancreatic hydatid cysts in Lebanon, adding valuable insight to the limited global literature on this unusual dual-organ involvement.

## Case presentation

A 71-year-old Lebanese woman presented with a six-month history of persistent, progressively worsening epigastric and right upper quadrant pain. She denied fever, vomiting, diarrhea, weight loss, smoking, alcohol use, recent travel, or animal contact. Her medical history included hypertension and atrial fibrillation, with no prior abdominal surgeries. Eight months earlier, she had undergone upper and lower endoscopy for similar complaints. Esophagogastroduodenoscopy (EGD) revealed a benign gastric ulcer with negative biopsy results, which was treated with proton pump inhibitors. Colonoscopy was unremarkable. Despite therapy, her symptoms persisted.

Six months later, she developed worsening abdominal pain, jaundice, and chills. Examination revealed scleral icterus and mild right upper quadrant tenderness. Laboratory studies showed leukocytosis with neutrophilia, markedly elevated transaminases, cholestatic enzyme elevation, hyperbilirubinemia, and elevated CRP, consistent with acute cholangitis. Amylase and lipase were normal (Table 1).

**Table 1:** Laboratory investigation of the patient during hospitalization. CRP: C-reactive protein; SGOT: serum glutamic-oxaloacetic transaminase; SGPT: serum glutamate-pyruvate transaminase; GGT: gamma-glutamyl transferase; Alk-Ph: alkaline phosphatase

Parameter	Patient value	Reference range
WBC	14,000/mm^3^	4,500-11,000/mm^3^
Hemoglobin	12.5 g/dL	13.5-17.5 g/dL
Platelets	172 x 10^9^/L	150-400 x 10^9^/L
Creatinine	0.7 mg/dL	<1 mg/dL
CRP	60 mg/dL	<5 mg/L
SGPT	300 IU/L	<35 IU/L
SGOT	340 IU/L	<50 IU/L
Alk-Ph	130 IU/L	<150 IU/L
GGT	107 IU/L	<65 IU/L
Amylase	65 IU/L	<35 IU/L
Lipase	37 IU/L	<70 IU/L
Total bilirubin	4.5 mg/dL	<1 mg/dL
Direct bilirubin	3 mg/dL	<0.2 mg/dL

**Figure 1:**
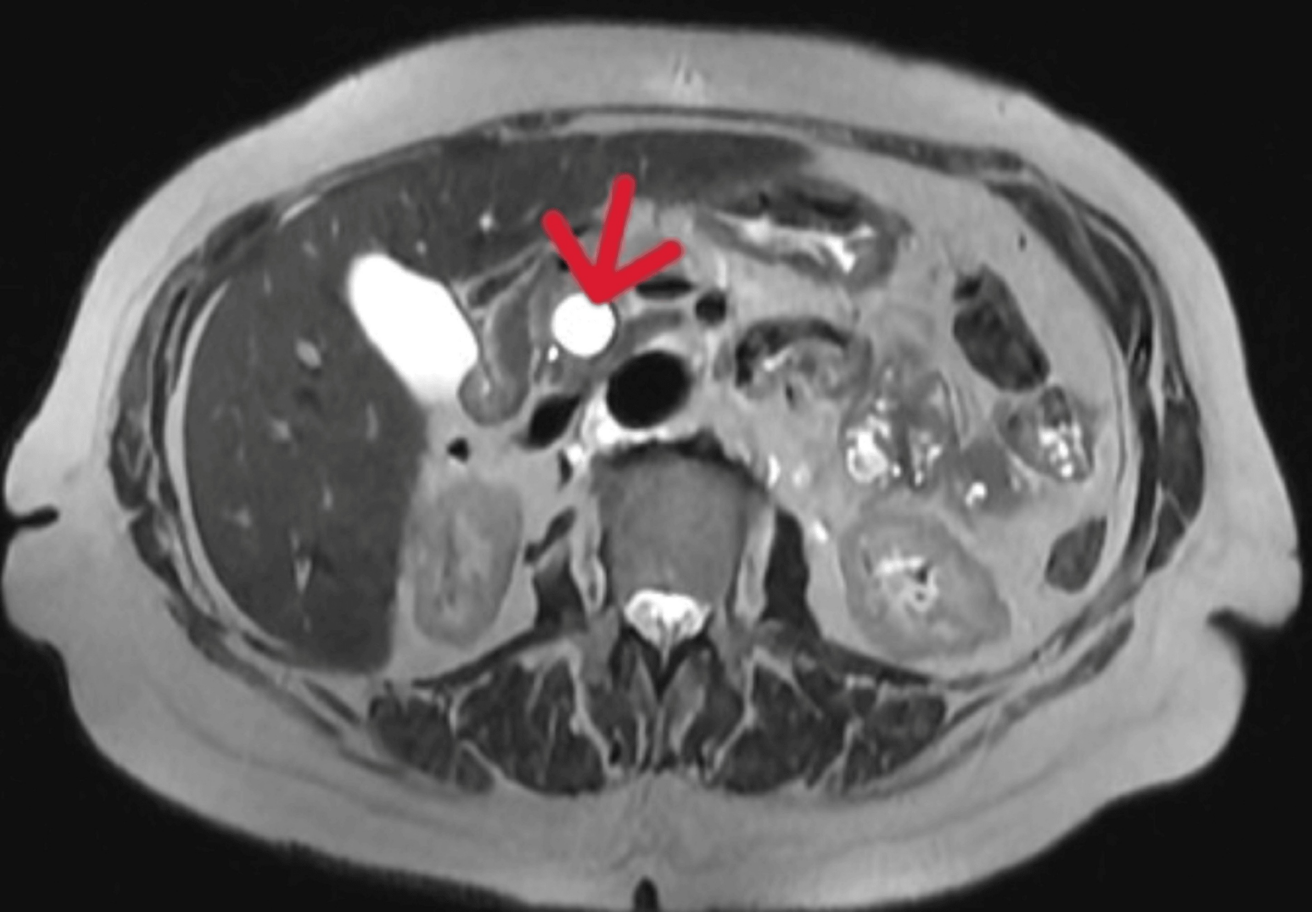
Axial MRI T2-weighted SSFSE sequence. The arrow indicates a well-defined cystic lesion in the pancreatic head. SSFSE: single-shot fast spin-echo

**Figure 2:**
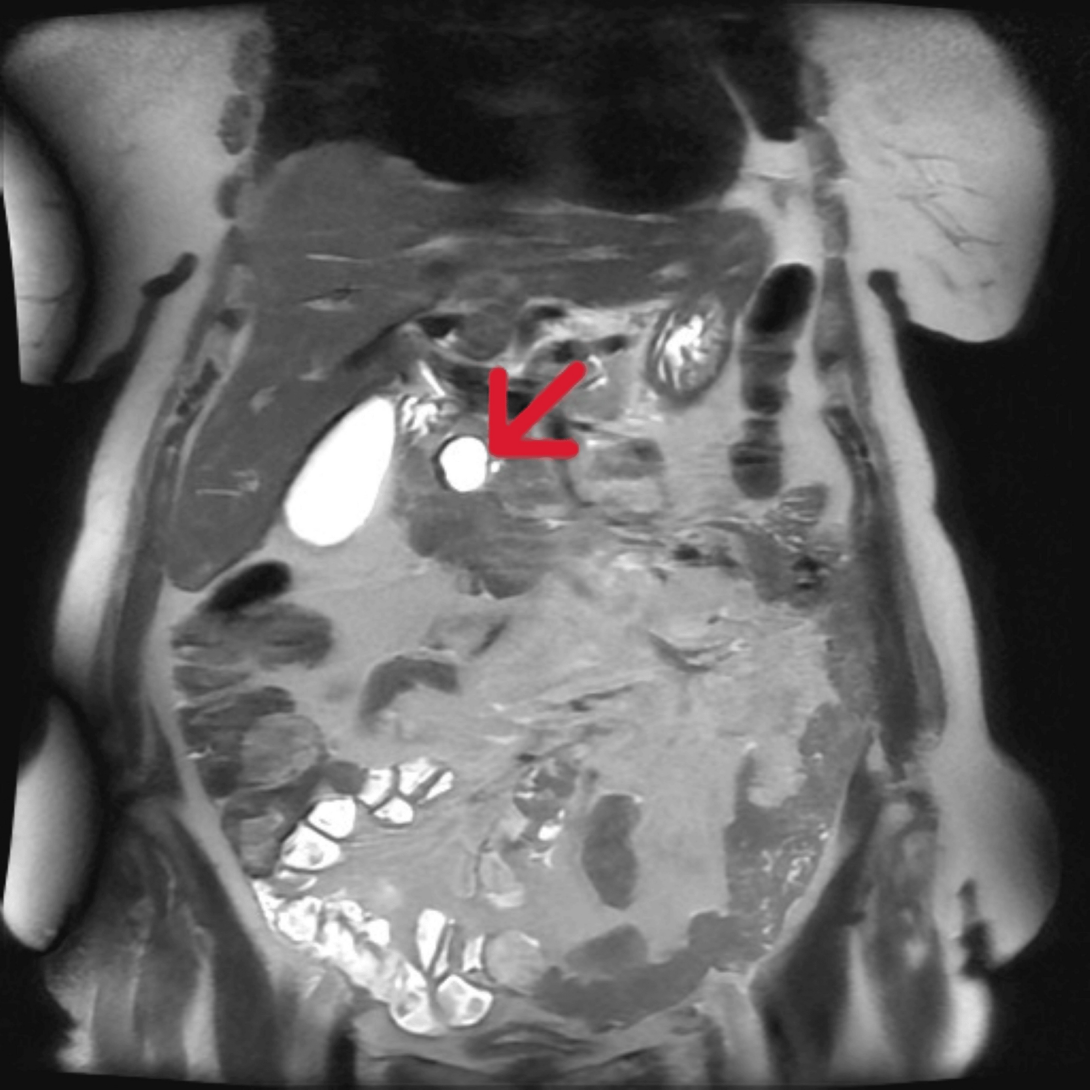
Coronal MRI T2-weighted SSFSE sequence of the abdomen. The image shows a well-defined cystic lesion in the pancreatic head (arrow). SSFSE: single-shot fast spin-echo

MRI of the abdomen with magnetic resonance cholangiopancreatography (MRCP), performed with and without gadolinium, demonstrated hepatomegaly (185 mm) with a smooth and homogeneous parenchyma, containing a biliary cyst measuring 36 × 21 mm in segment VII. A 16-mm cystic lesion was noted in the pancreatic head, associated with upstream dilation of the Wirsung duct (4 mm) (Figures 1, 2).

The gallbladder appeared normal with homogeneous content and no signs of acute inflammation, and there was no dilation of the common bile duct or intrahepatic bile ducts. The spleen, adrenal glands, and kidneys were unremarkable, with no cystic or solid lesions and no pelvicalyceal system dilatation. Additionally, a supraumbilical hernia containing fat and a small amount of fluid, as well as an infraumbilical hernia containing fat, were identified. MRCP confirmed the pancreatic head cyst with ductal dilatation and the hepatic cyst in segment VII (Video 1). 

**Video 1:** MRI T2-weighted SSFSE sequence of the abdomen. The video demonstrates a well-defined cystic lesion in the pancreatic head. SSFSE: single-shot fast spin-echo

The patient underwent an exploratory laparotomy, which revealed cystic lesions in the pancreatic head and liver. A Roux-en-Y cystojejunostomy was performed. Gross inspection of the cysts suggested hydatid disease, which was later confirmed histopathologically as *E. granulosus *infection. Postoperatively, the patient recovered without complications and was discharged on albendazole 400 mg twice daily for six months.

## Discussion

Pancreatic cysts encompass a broad spectrum from benign to malignant lesions, most of which are neoplastic or inflammatory in origin, including intraductal papillary mucinous neoplasms (IPMNs), mucinous cystic neoplasms (MCNs), solid pseudopapillary neoplasms (SPNs), and cystic pancreatic neuroendocrine tumors [1]. Most are detected incidentally during cross-sectional imaging (CT or MRI) performed for unrelated indications. When symptomatic, they may cause abdominal pain, pancreatitis, weight loss, or jaundice [3]. Reported prevalence varies widely, with imaging-based studies estimating rates between 2% and 20%, MRI-based studies reaching up to 38%, and an overall average of about 15% in the general population. Autopsy series have reported even higher rates (24-50%), with prevalence increasing with age [4].

While the majority of pancreatic cysts are neoplastic or inflammatory, rare parasitic causes should also be considered. Among these, pancreatic hydatid cysts represent an extremely uncommon manifestation of *E. granulosus* infection, a zoonosis transmitted from animals to humans. Humans acquire the infection through ingestion of parasite eggs, commonly via contaminated food, water, or direct contact with infected animals. The larvae most often lodge in the liver (50-70%) and lungs (20-30%), but can also involve the kidneys, spleen, central nervous system, or other sites [2]. Pancreatic hydatid cysts are exceptionally rare, even in endemic regions, accounting for less than 1% of all hydatid disease cases and 0.2-2% of pancreatic cystic lesions [5]. Hematogenous spread is the most widely accepted route of pancreatic involvement, although biliary migration, lymphatic spread, retroperitoneal extension, and venous dissemination have also been proposed [6,7].

Symptoms of pancreatic hydatid cysts vary according to cyst size and location, ranging from vague abdominal discomfort to obstructive jaundice or recurrent pancreatitis if the biliary tree is compressed [8]. On imaging, they may mimic mucinous cystadenomas, particularly when unilocular. However, the presence of daughter cysts, calcified walls, or synchronous hepatic lesions in a patient from an endemic region should raise suspicion for hydatid disease [9].

Diagnosis typically integrates imaging modalities, such as ultrasonography, CT, or MRI, which may reveal characteristic features including internal septations or daughter cysts, with serological assays detecting echinococcal antibodies [5]. Recurrence is uncommon after complete surgical removal but may occur with intraoperative spillage or incomplete excision [8]. Coexisting hepatic hydatid cysts are common, occurring in up to 50% of cases due to the liver’s role as the primary filter for larvae [10]. Prognosis is generally favorable with timely intervention, but delayed diagnosis can lead to rupture, infection, or spread to adjacent structures [11].

Our patient presented with progressive abdominal pain, jaundice, and cholangitis, along with imaging evidence of cystic lesions in both the pancreatic head and liver, initially misinterpreted as a mucinous cystadenoma. Progressive lesion growth, concomitant hepatic involvement, and epidemiologic context prompted consideration of hydatid disease. Surgical exploration confirmed cystic lesions in both sites, and histopathology established the diagnosis of *E. granulosus* infection. Differentiating hydatid cysts from mucinous cystadenomas can be challenging, as both may present as unilocular cystic lesions in the pancreatic head. However, the presence of daughter cysts, calcified walls, or concurrent hepatic involvement in a patient from an endemic region should prompt consideration of hydatid disease. In our patient, progressive cyst growth and a synchronous hepatic lesion were key clues leading to the correct diagnosis.

Surgery remains the cornerstone of management for pancreatic hydatid cysts, particularly in large, symptomatic, or anatomically challenging lesions [12]. For cysts in the pancreatic head, a conservative procedure such as Roux-en-Y cystojejunostomy can achieve drainage while preserving pancreatic parenchyma and minimizing morbidity [13]. Adjunctive antiparasitic therapy with albendazole (10-15 mg/kg/day, up to 400 mg twice daily) is recommended both preoperatively and postoperatively for 3-6 months to sterilize cysts and reduce recurrence [14]. Albendazole demonstrates superior absorption and cure rates compared to mebendazole [15]. World Health Organization (WHO) guidelines advise starting therapy days to weeks before surgery and continuing for at least one month postoperatively, with extended courses offering better outcomes without significantly increasing toxicity [14].

In the present case, the patient underwent Roux-en-Y cystojejunostomy with an uneventful recovery and was discharged on albendazole 400 mg twice daily for six months, in line with current recommendations. This combined surgical and medical approach is supported in the literature as an effective strategy for treating pancreatic hydatid cysts.

## Conclusions

Pancreatic hydatid disease is an exceptionally rare manifestation of *Echinococcus granulosus* infection, often presenting with nonspecific symptoms and imaging findings that closely resemble other cystic pancreatic neoplasms. This diagnostic challenge is particularly pronounced in the absence of hepatic or pulmonary involvement; however, the coexistence of hepatic cysts in an endemic setting should heighten clinical suspicion. Clinicians should maintain a high index of suspicion for hydatid disease when evaluating pancreatic cystic lesions in endemic regions, especially when imaging reveals concurrent hepatic pathology. Accurate preoperative diagnosis is essential to guide appropriate surgical planning and to avoid intraoperative spillage, which may lead to recurrence or anaphylaxis. Finally, optimal outcomes depend on an integrated approach, combining definitive surgical management tailored to cyst location and size with perioperative antiparasitic therapy, ensuring complete eradication and minimizing recurrence risk. Early recognition and coordinated surgical-medical management can achieve excellent outcomes, as demonstrated by our patient’s uneventful postoperative recovery.
